# Annotations

**Published:** 1890-07-05

**Authors:** 


### Annotations.

**About Cucumbers.**

Many people are under the impression that cucumber is very indigestible; and when they eat it they do so under protest, and with apprehensions of possibly dire consequences. How this delusion can have arisen it is difficult to say, unless it be that cucumber is often eaten with salmon and other indigestible table friends. It is not the cucumber, however, but the salmon, that sits so heavily upon our stomach's throne. Cucumber, in fact, is very digestible when eaten properly. It cannot indeed be otherwise when it is remembered that it consists mainly of water; and that those parts which are not water are almost exclusively cells of a very rapid growth. In eating cucumber it is well to cut it into thin slices, and to masticate them thoroughly. Even the vinegar and the pepper that are so often added to it are of service to digestion if not taken in excess. The cucumber, as everyone knows, belongs to the melon tribe; but in our somewhat cold country, it does not grow to any very large size, and therefore it is firmer and looks less digestible than its congener, the melon. Whilst on this topic, we may remind our readers that the time for abundance of fresh vegetables has now arrived, and that every one who wishes to be healthy should eat sparingly of meat and freely of the produce of the garden in the summer season. Vegetarianism, though somewhat of a fad, embodies a very important half truth. Many people undoubtedly eat a great deal too much meat. They thereby put a strain on digestion and encumber their lymphatic glands with waste matter of a decidedly dangerous kind. Fresh vegetables and fresh fruits increase the bulk of the contents of the alimentary canal and thus promote thoroughness of excretion. They, in fact, produce the effect of an improved sweeping-out apparatus, and so both lighten the body and diminish the work of certain important excretory glands. In the summer, when both cucumber and melon, and strawberry and all the produce of the garden is abundant, the meals should be varied as much as possible, and should consist, as we have said, principally of vegetables and fruit. The physic of the fields and the garden should always be preferred to the physic of the laboratory where it can be conveniently procured.

**Normandy Butter.**

The British Vice-Consul at Caen reports that certain butter dealers and merchants have again been very largely introducing the use of various fatty compounds for mixing with pure butter; and that in spite of special laws recently adopted in France against these fraudulent practices, this particular fraud has been, and still is, carried on to a large extent. The exporters have for some time past been sending to the English market "Normandy Butter" heavily adulterated with foreign fatty compounds. It appears that the export of Normandy butter in 1882 to the English market alone amounted to nearly ninety million francs; while in 1887 it had fallen to fifty-eight million francs, and that since the latter year there has been a still further decrease. We cannot but express our satisfaction that the butter makers and dealers who condescend to add cheating and thieving to their business are now being made to suffer the only kind of punishment which they really understand, viz., a diminution of their business and a loss of their profits. English people are not quite so stupid as the cynical Frenchmen have supposed. What we should like to see would be the entire disappearance of Normandy and all other foreign butters from the English market, as the result of an abundant and cheap home supply. Butter making at an average price of tenpence or a shilling a pound all the year round is a very profitable business in the present condition of the land and labour market. But if that be so, why are farmers generally so poor? The answer is, first, that they are not now generally so poor; and secondly, that where they are very poor, it is to a great extent their own fault. They are not men of business. They have been for centuries accustomed to do things in the gross, and have given no attention to details. Butter-making, for example, would be considered by many of them to entail a loss of dignity; and for a farmer himself to superintend the details of his own dairy would be to provoke the laughter of all his neighbours who should hear of the fact. Can anything be more preposterously silly than this state of mind? We defy all the philosophers of agriculture to show by argument that there is more dignity in superintending the growth of a field of wurzel than in superintending the manufacture of half-a-ton of butter. The truth is that farmers lack two things, education and capital, but education most of all. As a class, they are destitute of thinking power. If they were not, is it conceivable that they would allow three or four million sterling to be sent out of the country every year to one limited district of France, and for such an article of food as butter? Land in our own country is running to waste, and labour is starving for want of profitable employment, and yet millions upon millions of money are annually sent to foreign countries for butter, eggs, fruit, hops, corn, beef, and innumerable other products. Most of these millions could be kept at home if our farmers and landed proprietors were men of business and brains. Now that Normandy butter is in such bad repute with consumers, will not our own butter-makers, as a matter both of self-interest and of patriotism, do their utmost to bring back the butter-making industry to their own country?

**Tuberculous Cattle.**

We are not among those who take delight in publishing sensational paragraphs about dangerous meat, poisonous milk, diseased animals, and the like. But, inasmuch as public opinion is the all-powerful lever whereby we are all raised to a higher level of general sanitation and of personal health, we hold it a duty to lay important sanitary facts before our readers from time to time, and to do what is possible to show the true bearings and relative importance of those facts. Certain official statements relating to the public slaughter-house of Augsburg have been recently published, and demand general attention. It is not improbable that similar slaughter-houses in this country would furnish similar facts. During the last year, 1889, there were 23,592 calves slaughtered, and of these one only was found to be tuberculous. But among older cattle a very different state of things prevailed. Of 13,679 which were killed in the slaughter-house, no fewer than 612, or 4·4 per cent., were tuberculous. Those thirteen thousand odd consisted of 8,537 oxen, of which 167, or 1·94 per cent., were tuberculous; and of 5,008 cows, of which 445, or 8·88 per cent., were affected with tubercle. In four cases, or about one in every hundred of the cows, the udder was the part affected by the tuberculosis. In 67 of the 612 diseased animals the flesh was declared unfit for human food on account of generalized tuberculosis, and it was consequently destroyed. The questions that the public may ask are, first, what is the relative amount of danger in eating tuberculous meat; and, secondly, what is the risk of infection from drinking the milk of tuberculous cows? With regard to the first, the danger from the eating of tuberculous meat is very small, especially to the wealthier classes. The average British butcher is a man whose conscience is not very susceptible in the matter of profits; but he mostly has sufficient sense to refrain from supplying his customers with diseased meat. Moreover, there is no doubt whatever that when beef or other meat is sufficiently cooked, any tuberculous germs that may be in it are killed and rendered harmless. The simple rule of safety with regard to meat is to avoid eating it underdone. The danger arising from drinking tuberculous milk is probably much more considerable. This is particularly the case in large towns where many cattle are shut up in close sheds month after month, and never get a single breath of really pure air. In order to avoid as much as possible the risk of drinking tuberculous milk, it is well to purchase at those dairies which receive their supplies from the country. But the only way to really render milk always harmless is to cook it thoroughly, exactly as meat is cooked. The dangers which we have pointed out, although they may appear somewhat remote, are undoubtedly real; and the public will preserve its own health best by thoroughly understanding the subject, and keeping both butchers and milk-sellers intelligently in check.

